# Activation of the NLRP3 Inflammasome Pathway by Uropathogenic *Escherichia coli* Is Virulence Factor-Dependent and Influences Colonization of Bladder Epithelial Cells

**DOI:** 10.3389/fcimb.2018.00081

**Published:** 2018-03-14

**Authors:** Isak Demirel, Alexander Persson, Annelie Brauner, Eva Särndahl, Robert Kruse, Katarina Persson

**Affiliations:** ^1^iRiSC - Inflammatory Response and Infection Susceptibility Centre, Faculty of Medicine and Health, Örebro University, Örebro, Sweden; ^2^School of Medical Sciences, Örebro University, Örebro, Sweden; ^3^Department of Microbiology, Tumor and Cell Biology, Division of Clinical Microbiology, Karolinska Institutet and Karolinska University Hospital, Stockholm, Sweden; ^4^Department of Clinical Research Laboratory, Faculty of Medicine and Health, Örebro University, Örebro, Sweden

**Keywords:** UPEC, NLRP3 inflammasome, IL-1β, α-hemolysin, type-1 fimbriae

## Abstract

The NLRP3 inflammasome and IL-1β release have recently been suggested to be important for the progression of urinary tract infection (UTI). However, much is still unknown regarding the interaction of UPEC and the NLRP3 inflammasome. The purpose of this study was to elucidate what virulence factors uropathogenic *Escherichia coli* (UPEC) use to modulate NLRP3 inflammasome activation and subsequent IL-1β release and the role of NLRP3 for UPEC colonization of bladder epithelial cells. The bladder epithelial cell line 5637, CRISPR/Cas9 generated NLRP3, caspase-1 and mesotrypsin deficient cell lines and transformed primary bladder epithelial cells (HBLAK) were stimulated with UPEC isolates and the non-pathogenic MG1655 strain. We found that the UPEC strain CFT073, but not MG1655, induced an increased caspase-1 activity and IL-1β release from bladder epithelial cells. The increase was shown to be mediated by α-hemolysin activation of the NLRP3 inflammasome in an NF-κB-independent manner. The effect of α-hemolysin on IL-1β release was biphasic, initially suppressive, later inductive. Furthermore, the phase-locked type-1-fimbrial ON variant of CFT073 inhibited caspase-1 activation and IL-1β release. In addition, the ability of CFT073 to adhere to and invade NLRP3 deficient cells was significantly reduced compare to wild-type cells. The reduced colonization of NLRP3-deficient cells was type-1 fimbriae dependent. In conclusion, we found that the NLRP3 inflammasome was important for type-1 fimbriae-dependent colonization of bladder epithelial cells and that both type-1 fimbriae and α-hemolysin can modulate the activity of the NLRP3 inflammasome.

## Introduction

Urinary tract infection (UTI), the majority caused by uropathogenic *E. coli* (UPEC), is one of the most common human infections and 60% of all women are expected to report at least one episode of UTI during their lifetime. UPEC have been shown to persist in the urinary tract by the expression of several virulence factors that can manipulate the antibacterial host defenses (Bower et al., 2005; Yadav et al., 2010; Bien et al., 2012). Genomic analysis have identified considerable differences between UPEC isolates, making it difficult to pinpoint specific virulence factors associated with successful colonization of the urinary tract (Marrs et al., 2005; Lo et al., 2015). However, virulence factors such as lipopolysaccharide (LPS), toll/interleukin-1 receptor domain-containing protein (TcpC), siderophores (iron scavenger system), α-hemolysin, type-1-and P-fimbriae and capsular have been shown to play a role in the infection during a UTI (Bower et al., 2005; Yadav et al., 2010; Bien et al., 2012). The type-1 fimbriae is a key virulence factor that facilitates bacterial attachment to the bladder epithelium and enables thereby UPEC to resist being rinsed out by the urine flow. Furthermore, type-1 fimbriae also mediates invasion of bladder epithelial cells and modulation of mucosal inflammation (Martinez et al., 2000; Eto et al., 2007; Dhakal et al., 2008; Bien et al., 2012; Flores-Mireles et al., 2015). The pore-forming toxin α-hemolysin has been shown to have dual effects on urothelial cells depending on concentration. At low concentrations, α-hemolysin has a more immunomodulating effect and promotes exfoliation of bladder epithelial cells, whereas at high concentration, the toxin lyses epithelial and immune cells which enables UPEC to access nutrients and iron from host cells (Dhakal and Mulvey, 2012; Ristow and Welch, 2016). Hence, it is the interplay of several virulence factors that makes UPEC a successful colonizer of the urinary tract. Several studies have shown that UPEC can invade, replicate and form intracellular bacterial communities in bladder epithelial cells and that the majority of clinical UPEC isolates have this ability (Rosen et al., 2007; Hannan et al., 2012). Intracellular reservoirs can persist for several weeks, protected from antibiotics and host immune responses as a quiescent reservoir and efflux out from the intracellular niche and re-infect the bladder epithelium (Rosen et al., 2007; Hannan et al., 2012; Scott et al., 2015). After antibiotic treatment, approximately 25% of patients with UTI will have a recurring UTI within 6 months and 45% within 1 year (Bower et al., 2005; Yadav et al., 2010; Bien et al., 2012). Hence, the ability of UPEC to form protective intracellular reservoirs has been associated with host evasion and recurrent UTI (Rosen et al., 2007; Andersen et al., 2012; Hannan et al., 2012).

The immune response to an UPEC infection, primarily mediated by urothelial cells and neutrophils, strongly influences the clearance and outcome of the infection (Flores-Mireles et al., 2015). The role of pro-inflammatory cytokines, such as IL-6 and IL-8, during a UTI is well studied but more knowledge is needed on host immune factors that control and modulate UPEC colonization, particularly host factors that affect the intracellular localization of UPEC (Khalil et al., 2000) (Benson et al., 1996; Godaly et al., 2000). Inflammasomes are cytosolic multiprotein complexes that detect extra- and intracellular pathogens and/or danger signals, and activate caspase-1, which leads to caspase-1-dependent cell death (pyroptosis) or the maturation and release of pro-inflammatory cytokines, e.g., IL-1β and IL-18. The activation of the NLRP3 inflammasome usually requires two signals. The initial priming step affects NLRP3 and IL-1β at the transcription level and signal two promotes the assembly of the NLRP3 inflammasome and caspase-1 activation (Martinon et al., 2002; Broz and Dixit, 2016). The NACHT leucin-rich repeat PYD protein 3 (NLRP3) inflammasome has recently been emphasized to play an important role in the progression of UTI (Nagamatsu et al., 2015; Symington et al., 2015; Ambite et al., 2016). However, the role of NLRP3 in UPEC colonization of bladder epithelial cells has previously not been investigated.

Nagamatsu and colleagues showed that UPEC α-hemolysin induced caspase-1/caspase-4-dependent cell death in bladder urothelial cells, which was associated with an increased IL-1β release (Nagamatsu et al., 2015). In addition, IL-1β was recently shown to be important for the outcome of UTI (Ambite et al., 2016; Waldhuber et al., 2016). The destruction of intracellular bacterial replication niches and the release of inflammatory cytokines may represent key mechanisms by which inflammasome activation and pyroptosis may contribute to the immune response in the urinary tract. The aim of this study was to elucidate the virulence factors used by UPEC to modulate NLRP3 inflammasome activation and IL-1β release, and the role of NLRP3 for UPEC colonization of human bladder epithelial cells.

## Materials and methods

### Human bladder epithelial cells

The human bladder epithelial cell line 5637 (ATCC HTB-9) was obtained from the American Type Culture Collection (Manassas, VA, USA). Cells were grown in Dulbecco's Modified Eagle Medium (DMEM) (Lonza, Basel, Switzerland) supplemented with 10% fetal bovine serum (FBS), 2 mM L-glutamine, 1 mM non-essential amino acids (all from Thermo Fisher Scientific, Waltham, MA, USA) at 37°C in a 5% CO_2_ atmosphere and subcultured when confluent. During experiments, the cell medium was replaced with DMEM containing 2% FBS, 1 mM non-essential amino acids and 2 mM L-glutamine. The human bladder epithelial cell line HBLAK (CELLnTEC Advanced Cell Systems AG, Bern, Switzerland) has been isolated from a healthy bladder and been spontaneously transformed providing the convenience of long-term cell growth without senescence. HBLAK cells were cultured in CnT-57 cell culture medium (CELLnTEC) supplemented with 50 U/mL penicillin and 50 μg/ml streptomycin (both from Thermo Fisher Scientific) in a humidified atmosphere with 5% CO_2_ at 37°C. The cell medium was replaced with RPMI 1640 (Lonza) containing 2% FBS, 1 mM non-essential amino acids and 2 mM L-glutamine 24 h prior to experiment and the same medium was used during experiments.

### CRISPR/Cas9 gene editing

5,637 bladder epithelial cells were seeded (100,000 cells/well) in a 24-well plate and grown in DMEM supplemented with 10% FBS, 2 mM L-glutamine, 1 mM non-essential amino acids for 24 h at 37°C with 5% CO_2._ The Cas9 and gRNA expressing plasmid pSpCas9 (BB)-2A-Puro (PX459) V2.0 (gift from Feng Zhang, Addgene plasmid #62988) (Ran et al., 2013) was used for gene editing. The CRISPR target sites were: GCTAATGATCGACTTCAATG (NLRP3), TTATCCGTTCCATGGGTGA (Caspase-1, gRNA1), GACAGTATTCCTAGAAGAAC (Caspase-1 gRNA2) and GAGTGTCCCTGTTGTATTTA (Mesotrypsin). 5637 cells were transfected with 500 ng plasmid and 1.5 μl of Lipofectamine 2000 in Opti-MEM (Thermo Fisher Scientific) according to manufacturer's instructions. The transfection was stopped after 6 h by replacing the medium with DMEM supplemented with 10% FBS, 2 mM L-glutamine, 1 mM non-essential amino acids. The cells were selected 24 h after transfection with puromycin (2.5 μg/ml, Sigma-Aldrich, St. Louis, MO, USA) for 24 h. All experiments were conducted with a polyclonal pool of gene edited cells. The phenotype was confirmed by Western blot.

### Bacterial isolates, plasmids and gene cloning

Four hemolysin positive and four hemolysin negative UPEC isolates were obtained from the Department of Microbiology at Örebro University hospital, Sweden, from anonymized individuals with suspected pyelonephritis. MG1655, a non-pathogenic *E. coli* K-12 strain, CFT073, a UPEC strain isolated from an individual with pyelonephritis and ESBL019, a UPEC isolate isolated from an individual with urosepsis (Demirel et al., 2017) were used in this study. CFT073 deletion mutants CFT073ΔfimH and CFT073ΔhlyA were created using λ Red Recombinase (Datsenko and Wanner, 2000) using primer sets *hlyA*_Fwd 5′-AAAAACAAGACAGATTTCAATTTTTCATTAACAGGTTAAGAGATAATTAAGTGTAGGCTGGAGCTGCTTC-′3 and *hlyA*_Rev 5′-AATCTTATGTGGCACAGCCCAGTAAGATTGCTATTATTTAAATTAATAAAATGGGAATTAGCCATGGTCC-′3 and primer sets *fimH*_Fwd 5′-CATTCAGGCAGTGATTAGCATCACCTATACCTACAGCTGAACCCAAAGAGGTGTAGGCTGGAGCTGCTTC-′3 and *fimH*_Rev 5′-TAGCTTCAGGTAATATTGCGTACCTGCATTAGCAATGCCCTGTGATTTCTATGGGAATTAGCCATGGTCC-′3, respectively (Thermo Fisher Scientific). The mutants CFT073Δpap (Mobley et al., 1993) and CFT073 fim L-ON (Gunther et al., 2002) were a kind gift from Professor Harry Mobley at University of Michigan Medical School, MI, USA. The hlyCARD expression plasmid pGNH404 was a kind gift from Professor Agneta Richter-Dahlfors at Karolinska Institute, Solna, Sweden. The eGFP expressing pLMB449 plasmid was a kind gift from Professor Philip Poole at University of Oxford, Oxford, UK. The bacteria were maintained on tryptic soy agar (TSA) and cultured overnight in Luria broth (LB) (Lennox; Franklin Lakes, NJ, USA) at 37°C aerobically on a shaker. Bacteria were suspended in phosphate buffered saline (PBS) to the appropriate concentrations. Hemolytic activity was assessed on blood agar plates.

### Cell stimulation procedures

The bladder epithelial cell line 5637 (wild type: Cas9) and the caspase-1 -NLRP3 -and mesotrypsin-deficient cells were stimulated with different UPEC isolates, CFT073 mutant bacteria or the commensal *E. coli* MG1655 for 3 or 6 h at a multiplicity of infection (MOI) of 10 and incubated at 37°C with 5% CO_2_. Supernatants, mRNA and protein were collected and kept at−80°C until further analysis. Bladder epithelial cells were also pre-incubated with DMSO (vehicle), caspase-1/4 inhibitor Ac-YVAD-CHO (10 μM, Enzo Life Sciences, NY, USA), caspase-3 inhibitor Ac-DEVD-CHO (10 μM, Enzo Life Sciences), JNK inhibitor SP600125 (10 μM, InSolutionT M JNK Inhibitor II, Calbiochem, USA), p38 MAPK inhibitor SB203580 (10 μM, Santa Cruz Biotechnology Inc., Heidelberg, Germany), ERK1/2 inhibitor PD98059 (10 μM, Santa Cruz Biotechnology Inc.), NF-κB inhibitor BAY 11-7082 (5 μM, Enzo Life Sciences), reactive oxygen species (ROS) inhibitor diphenyleneiodonium chloride (DPI, 10 μM, Santa Cruz Biotechnology Inc.), serine protease inhibitor 3,4-dichloroisocoumarin (DCI, 20 or 100 μM, Merck Millipore, MA, USA) for 1 h prior to CFT073 stimulation for 6 h at MOI 1 or 10. All inhibitors were optimized by concentration response and cell viability studies. The bladder epithelial cell line HBLAK was stimulated with CFT073 and the CFT073 mutants for 6 h at MOI 10 and incubated at 37°C with 5% CO_2_.

### RNA isolation and real time RT-PCR

Total RNA was isolated from bladder epithelial cells using E.Z.N.A. ® Total RNA Kit I (Omega Bio-tek, GA, USA) according to manufacturer's protocol. The concentration of RNA was determined using spectrophotometry (Nano- Drop ND-1000, Wilmington, NC, USA). First strand cDNA synthesis was performed by High capacity cDNA RT kit (Thermo Fisher Scientific). The real time-RT-PCR was carried out in a total volume of 10 μl on a 96-well plate (Sarstedt, Nümbrecht, Germany). Each well contained 5 μl Maxima SYBR Green qPCR Master Mix (Thermofisher), 1 μl of each primer IL-1β (Forward: 5′-CCACAGACCTTCCAGGAGAATG-3, Revers: 5′-GTGCAGTTCAGTGATCGTACAGG-3′), NLRP3 (Forward: 5′-GGACTGAAGCACCTGTTGTGCA-3, Revers: 5′-TCCTGAGTCTCCCAAGGCATTC-3′), pro-caspase-1 (Forward: 5′-GCTGAGGTTGACATCACAGGCA-3, Revers: 5′-TGCTGTCAGAGGTCTTGTGCTC-3′), ASC (Forward: 5′-AGCTCACCGCTAACGTGCTGC-3, Revers: 5′-GCTTGGCTGCCGACTGAGGAG-3′), uroplakin 1a (Forward: 5′-CACCAAGCAGATGCTGACCTTC−3, Revers: 5′-GGACCAGATGTGCCACAGCATT-3′), uroplakin 3a (Forward: 5′-CTCACAGATCCTGAATGCCTACC-3, Revers: 5′-CCGTGGACATATTGACCAGGAC-3′), integrin α3 (Forward: 5′-GCCTGACAACAAGTGTGAGAGC-3, Revers: 5′-GGTGTTCGTCACGTTGATGCTC-3′), integrin β1 (Forward: 5′-GGATTCTCCAGAAGGTGGTTTCG-3, Revers: 5′-TGCCACCAAGTTTCCCATCTCC-3′), and glyceraldehyde 3-phosphate dehydrogenase (GAPDH) (Forward: 5′-GTCTCCTCTGACTTCAACAGCG-3, Revers: 5′-ACCACCCTGTTGCTGTAGCCAA-3′) (Eurofins MWG Synthesis GmbH, Ebersberg, Munich), 1 μl cDNA (10 ng) and 3 μl RNase free water. The PCR amplification was conducted in a CFX96 Touch™ Real-Time PCR Detection System (Biorad, Hercules, CA, USA) using the following protocol: initial denaturation at 95°C for 10 min, 40 cycles of denaturation at 95°C for 15 s followed by annealing/extension at 60°C for 60 s. Each PCR was followed by a dissociation curve analysis between 60-95°C. The Ct values were analyzed by the comparative Ct (ΔΔCt) method and normalized to the endogenous control GAPDH. Fold difference was calculated as 2^−ΔΔCt^.

### Cytokine and cell viability measurements

Supernatants were collected after bacterial stimulation of bladder epithelial cells and centrifuged for 5 min at 5,000 g and stored at −80°C. An enzyme-linked immunosorbent assay (ELISA) was performed to measure the IL-1β release from the bladder epithelial cells. The cytokine was measured using human IL-1β kits (ELISA MAX™ Deluxe Sets, BioLegend, San Diego, CA, USA) according to the manufacturer's instructions and measured on a spectrophotometer (Multiskan Ascent, Thermo Labsystems, Helsinki, Finland). Cell viability was assessed by Pierce LDH cytotoxicity assay (Thermo Fisher Scientific) and neutral red toxicity assay (Sigma-Aldrich) according to the manufacturer's instructions.

### Caspase-1 activity assay

Bladder epithelial cells were grown to confluence in a 96-well plate and pre-incubated with the caspase-1 substrate Ac-YVAD-AMC (Enzo Life Sciences, New York, NY, USA) for 1 h at 37°C 5% CO_2_. Thereafter, the cells were stimulated with MOI 10 of the different strains of *E. coli*. Samples were analyzed after 6 h with a fluorescent plate reader at excitation/emission settings of 340/440 nm (Fluostar Optima, BMG Labtech, Ortenberg, Germany).

### Western blot analysis

Cell supernatants were precipitated with 10% Trichloroacetic acid (TCA, Merck Millipore) for 1 h on ice followed by centrifugation and acetone wash before the pellet was re-suspended in 4x Laemmli buffer (Sigma-Aldrich) and boiled for 10 min in 95°C. Furthermore, bladder epithelial cells were harvested in RIPA buffer supplemented with Halt protease and phosphatase inhibitor cocktail (Thermo Fisher Scientific). The cells were homogenized with a syringe and needle. The DC protein assay (Bio-Rad Laboratories, Hercules, CA, USA) was used to measure the protein concentration of the samples. Equal amounts of protein were mixed with Laemmli buffer and boiled for 5 min at 95°C. The samples (10 μg or total supernatant precipitate) were subjected to 4-20% SDS-polyacrylamine gel electrophoresis and transferred to a polyvinylidene fluoride (PVDF) membrane (Bio-Rad Laboratories). The PVDF membrane was blocked with 3% BSA for 1 h. Caspase-1 protein was detected using a mouse monoclonal (AdipoGen Life Sciences, Buckingham, UK) against human caspase-1. NLRP3 was detected using a mouse monoclonal (Abnova, Taipei City, Taiwan) against human NLRP3. GAPDH was detected with a rabbit polyclonal antibody (Santa Cruz Biotechnology Inc, Heidelberg, Germany). All primary antibodies were incubated overnight. As secondary antibodies, goat anti rabbit IgG (HRP) (Abcam, Cambridge, UK) and goat anti mouse IgG (HRP) (Abcam) were used and incubated for 1 h at room temperature. The bands were imaged using Luminata Forte Western HRP Substrate (Merck Millipore, Darmstadt, Germany).

### Colonization and invasion assay

Bladder epithelial cells were infected with CFT073 (eGFP, enhanced green fluorescence protein), CFT073 fim L-ON (eGFP) or ESBL019 (eGFP) at MOI 1 or 10 at 37°C with 5% CO_2_ for 4 h to measure colonization. In some experiments, NLRP3-deficient and Cas9 cells were pre-incubated with 500 pg/ml IL-1β (Sigma-Aldrich) for 1 h prior to infection. The wells were then washed 10 times with PBS and the adhered/invaded (referred to as colonized) eGFP expressing bacteria were quantified and imaged with a Cytation 3 plate reader (BioTek, Winooski, VT, USA). Intracellular presence of bacteria was assessed by infecting bladder epithelial cells with CFT073 at MOI 1 and 10 for 2 h at 37°C with 5% CO_2_. The wells were washed 10 times with PBS after infection and the medium was replaced with DMEM 2% FBS supplemented with 100 μg/ml gentamicin and incubated for additional 2 h. The cells were thereafter washed three times with PBS and lysed with 0.1% Triton-x 100 in PBS. The intracellular bacteria were plated on TSA plates, incubated at 37°C overnight and CFU was counted.

### Data analysis

Data are expressed as mean ± SEM. Differences between groups were assessed by one-way ANOVA followed by Bonferroni multiple testing correction. Results were considered statistically significant at *p* < 0.05. *n* = number of independent biological experiments.

## Results

### The UPEC-strain CFT073 induces caspase-1 activation and IL-1β release

**Figure 1:**
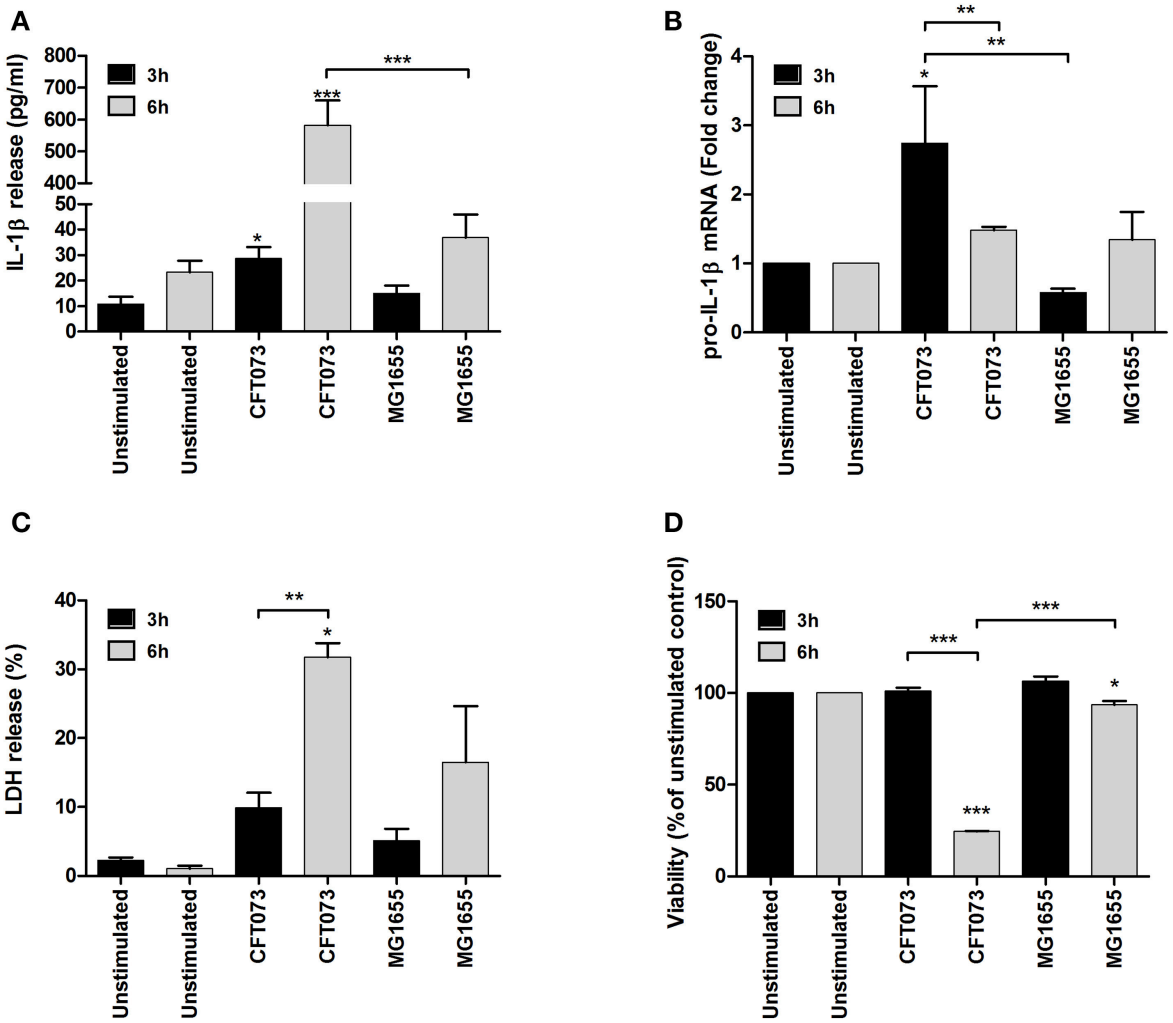
IL-1β release and cell viability analysis. The bladder epithelial cell line 5637 was infected with UPEC strain CFT073 and the non-pathogenic *E. coli* strain MG1655 at MOI 10 for 3 and 6 h followed by analysis of IL-1β release **(A)**, pro-IL-1β mRNA expression **(B)**, LDH release **(C)** and neutral red staining **(D)**. Pro-IL-1β expression was normalized to GAPDH and expressed as fold change relative to unstimulated controls. LDH release is presented as % of total LDH and neutral red as % of unstimulated controls. Data are presented as mean ± SEM (*n* = 3 independent experiments). Asterisks denote statistical significance compared to respective unstimulated control (*p < 0.05, **p < 0.01, ***p < 0.001).

The bladder epithelial cell line 5637 was stimulated with UPEC strain CFT073 and the non-pathogenic *E. coli* strain MG1655, and IL-1β release, caspase-1 activation and cell viability were assessed. The UPEC strain CFT073 induced a significant increase in IL-1β release from bladder epithelial cells compared to unstimulated cells after 6 h (Figure 1A), whereas MG1655 did not (Figure 1A). In addition, CFT073, but not MG1655 increased the mRNA expression of IL-1β compared to unstimulated cells (Figure 1B). However, neither CFT073 nor MG1655 increased mRNA expression of NLRP3, pro-caspase-1 or ASC (Figure S1). Evaluation of cell viability showed a significant increase in LDH release from CFT073-stimulated, but not MG1655-stimulated cells compared to unstimulated cells after 6, but not 3 h (Figure 1C). These findings were also validated with the neutral red viability assay measuring metabolic cell activity (Figure 1D).

**Figure 2:**
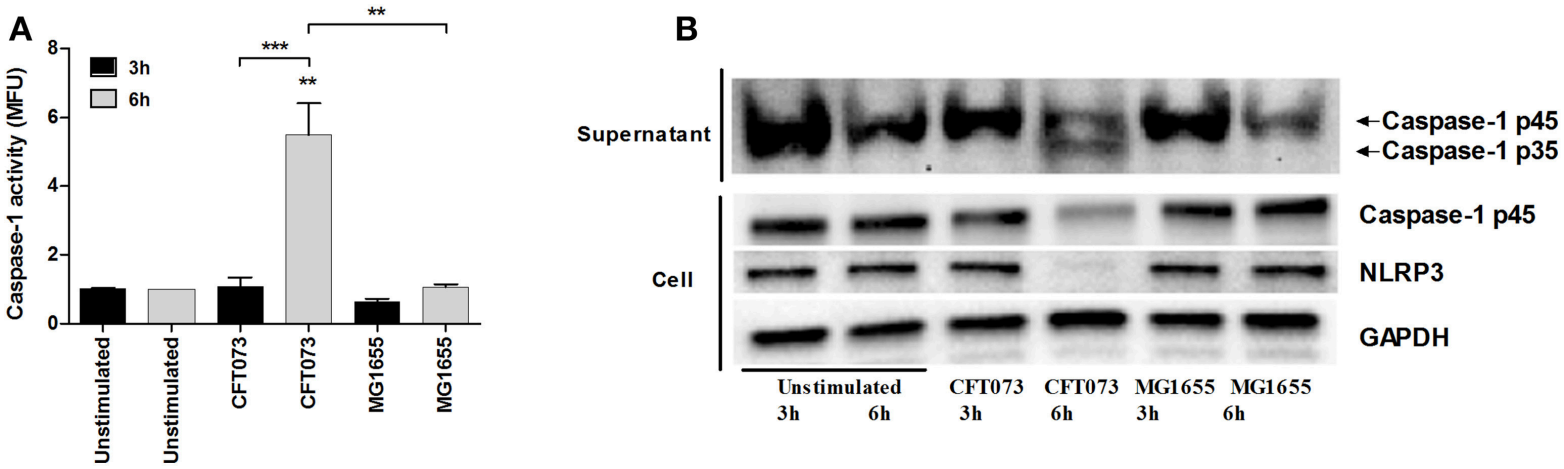
Caspase-1 activity and protein expression of caspase-1 and NLRP3. The bladder epithelial cell line 5637 was infected with UPEC strain CFT073 and the non-pathogenic *E. coli* strain MG1655 at MOI 10 for 3 and 6 h followed by analysis of caspase-1 activity **(A)** and caspase-1 and NLRP3 protein expression by Western blot analysis **(B)**. Caspase-1 activity is presented as fold increase of mean fluorescence units (MFU) compared to unstimulated control cells. GAPDH was used as a loading control for Western blots. Data are presented as mean ± SEM (*n* = 3 independent experiments). Asterisks denote statistical significance compared to respective unstimulated control (**p < 0.01, ***p < 0.001).

We next evaluated the ability of CFT073 and MG1655 to activate caspase-1 in bladder epithelial cells using a caspase-1 substrate assay. CFT073, but not MG1655, induced a significant increased caspase-1 activation compared to unstimulated cells after 6, but not 3 h (Figure 2A). Western blot analysis detected the processed intermediate p35 form of caspase-1 in the cell supernatant upon CFT073 stimulation for 6 h (Figure 2B). In addition, protein levels of NLRP3 were markedly decreased after CFT073 stimulation for 6, but not 3 h (Figure 2B). These findings show that the uropathogenic strain CFT073, but not the non-pathogenic MG1655 strain, induces caspase-1 activation and IL-1β release from bladder epithelial cells.

### α-hemolysin and type-1 fimbriae modulate caspase-1 activation and IL-1β release

**Figure 3:**
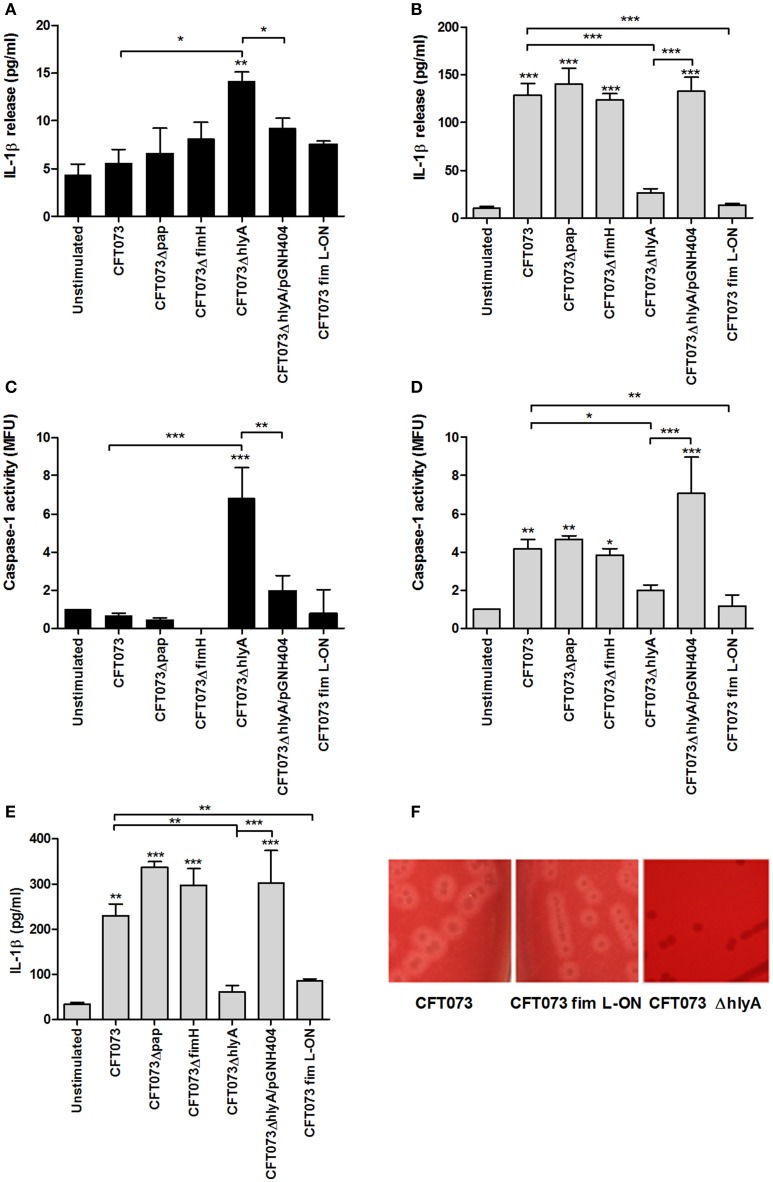
The importance of different virulence factors for IL-1β release and caspase-1 activity. The bladder epithelial cell line 5637 **(A–D)** and a spontaneously transformed bladder epithelial cell line HBLAK **(E)** were infected with CFT073, CFT073Δpap, CFT073ΔfimH, CFT073ΔhlyA, CFT073ΔhlyA/pGNH404 and CFT073 fim L-ON at MOI 10 for 3 **(A,C)** and 6 h **(B,D,E)**. IL-1β release **(A,B,E)** and caspase-1 activity **(C,D)** were measured. Caspase-1 results are presented as fold increase of mean fluorescence units (MFU) compared to unstimulated control cells. Hemolysin activity on blood agar was evaluated for CFT073, CFT073 fim L-ON, and CFT073ΔhlyA after overnight incubation **(F)**. Data are presented as mean ± SEM (*n* = 3 independent experiments). Asterisks denote statistical significance compared to respective unstimulated control (*p < 0.05, **p < 0.01, ***p < 0.001).

Experiments were performed to examine the involvement of various UPEC virulence factors on caspase-1 activation and IL-1β release using P-fimbriae (*pap*), type-1 fimbriae (*fimH*) and α-hemolysin (*hlyA*) CFT073 deletion mutants as well as a phase-locked type-1 fimbrial ON variant of CFT073 (CFT073 fim L-ON). These experiments showed that the Δ*hlyA* deletion mutant induced a significant increase in IL-1β release (Figure 3A) and caspase-1 activation (Figure 3C) compared to unstimulated cells, the wild-type CFT073 and to the hemolysin complemented CFT073ΔhlyA/pGNH404 strain after 3 h. This was not due to altered cell viability (Figure S2). However, after 6 h of stimulation, Δ*hlyA* induced a significant lower IL-1β release (Figure 3B) and caspase-1 activation (Figure 3D) compared to the wild-type CFT073 and the α-hemolysin complemented CFT073ΔhlyA/pGNH404 strain. The Δ*pap* and Δ*fimH* deletion mutants did however not show any differences in caspase-1 activation and IL-1β release compared to the wild-type CFT073. Notable, the CFT073 fim L-ON strain induced a significantly lower IL-1β release (Figure 3B) and caspase-1 activation (Figure 3D) compared to the wild-type CFT073. Furthermore, both CFT073 and the CFT073 fim L-ON strain showed hemolytic activity on blood agar (Figure 3F). These data suggest that the lower IL-1β release induced by CFT073 fim L-ON is α-hemolysin independent.

The IL-1β release experiments performed in the bladder cell line 5637 was repeated and validated using the spontaneously transformed bladder epithelial cell line HBLAK. The Δ*hlyA* and CFT073 fim L-ON strain induced a significantly lower IL-1β release compared to the wild-type CFT073 stimulated cells after 6 h (Figure 3E); in agreement with our findings from the 5637 cell line. These results confirm that both α-hemolysin and type-1 fimbriae can modulate caspase-1 activation and IL-1β release from bladder epithelial cells.

### Clinical α-hemolysin positive UPEC isolates induce caspase-1 activation and IL-1β release

**Figure 4:**
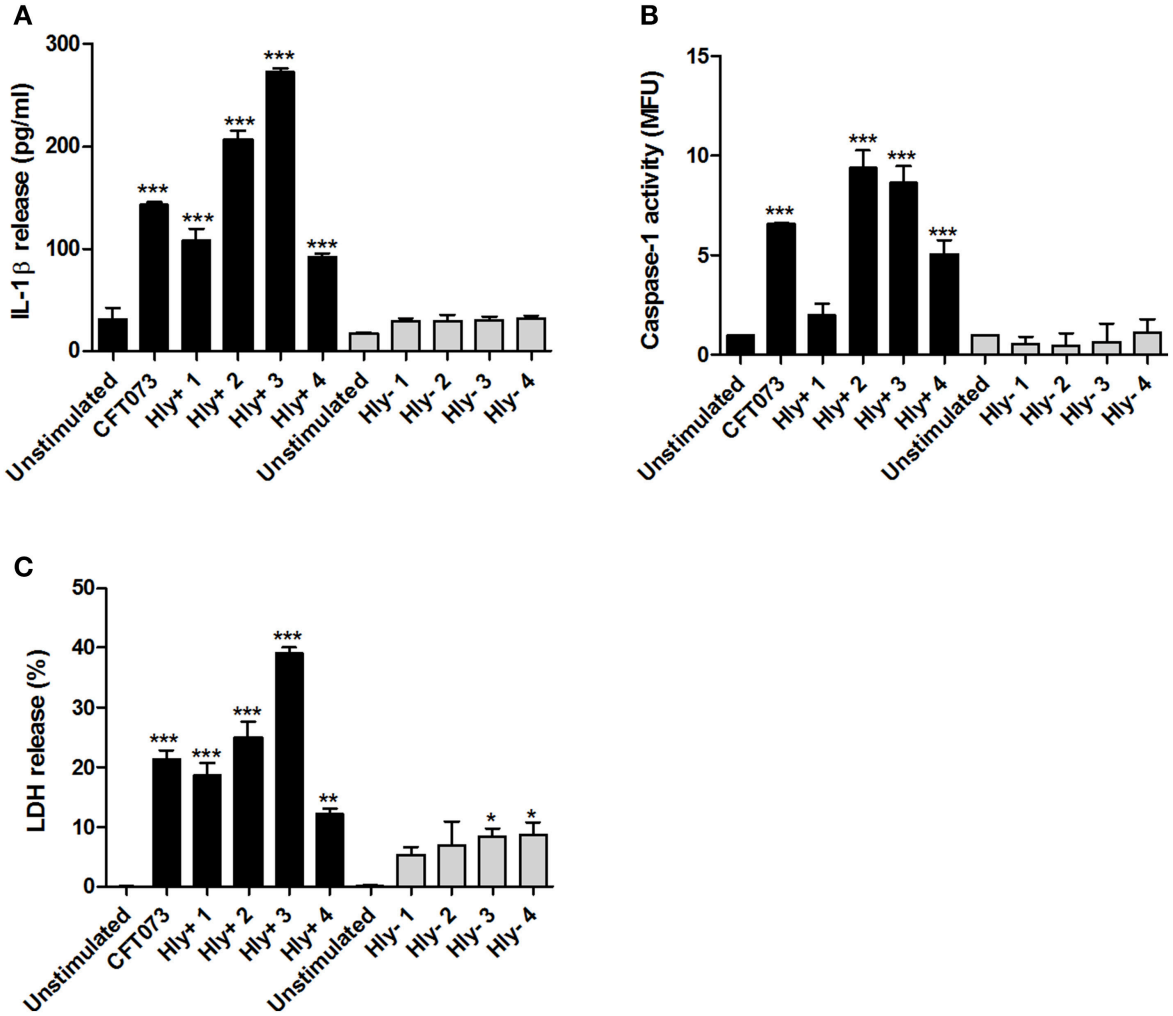
IL-1β release, caspase-1 activity and LDH release in α-hemolysin positive or negative clinical UPEC isolates. The bladder epithelial cell line 5637 was infected with CFT073 and four α-hemolysin positive UPEC isolates (Hly+) and four α-hemolysin negative UPEC isolates (Hly-) for 6 h at MOI 10. IL-1β release **(A)**, caspase-1 activity **(B)** and LDH release **(C)** were evaluated. LDH release is presented as % of total LDH. Data are presented as mean ± SEM (*n* = 3 independent experiments). Asterisks denote statistical significance compared to respective unstimulated control (*p < 0.05, **p < 0.01, ***p < 0.001).

We continued by validating the involvement of α-hemolysin using eight clinical UPEC isolates that were either α-hemolysin positive or α-hemolysin negative. The α-hemolysin positive isolates, but not the negative, induced a significant increase in IL-1β release (Figure 4A) and caspase-1 activation (Figure 4B) after 6 h compared to unstimulated cells. In addition, we found that the α-hemolysin positive isolates induced a higher LDH release compared to α-hemolysin negative isolates (Figure 4C). These results strengthen the notion that α-hemolysin is an important virulence factor for caspase-1 activation and IL-1β release from host bladder epithelial cells.

### α-hemolysin-induced IL-1β release and cell death is caspase-1 and NLRP3 dependent

**Figure 5:**
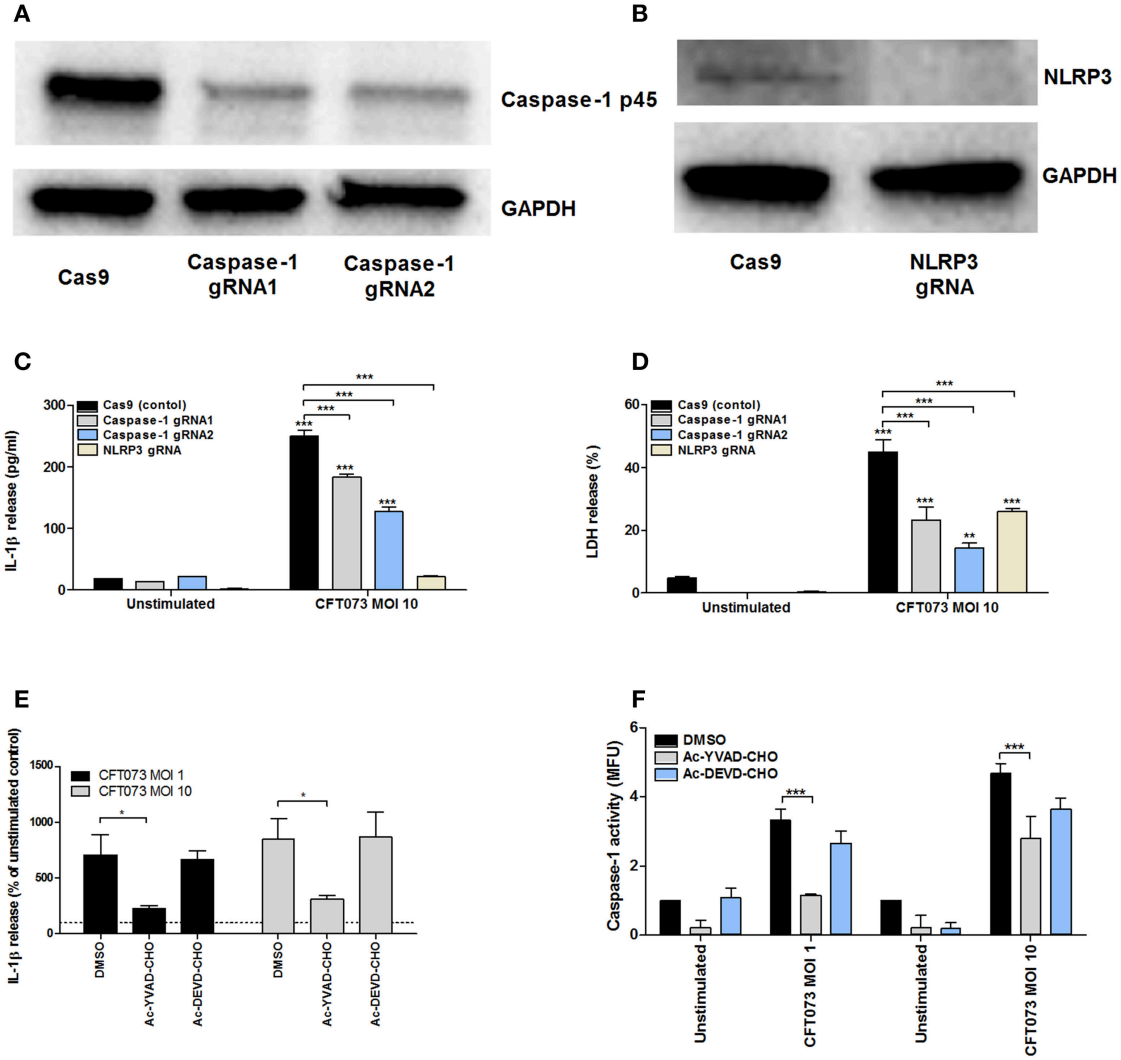
Analysis of IL-1β release, caspase-1 activity and LDH release in caspase-1 or NLRP3 deficient 5637 cells. Caspase-1 **(A)** and NLRP3 **(B)** knockdowns were created by CRISPR/Cas9 and evaluated by Western blot. GAPDH was used as a loading control. Cas9 (control cells), -caspase-1, or NLRP3-deficient bladder epithelial 5637 cells were infected with CFT073 for 6 h at MOI 10. IL-1β **(C)** and LDH release **(D)** were measured. 5637 cells were pre-incubated with DMSO, the caspase 1/4 inhibitor AC-YVAD-CHO (10 μM) or the caspase 3 inhibitor AC-DEVD-CHO (10 μM) for 1 h prior to infection with CFT073 at MOI 1 and 10 for 6 h followed by analysis of IL-1β release **(E)** and caspase-1 activity **(F)**. Caspase-1 results are presented as fold increase of mean fluorescence units (MFU) compared to unstimulated control cells. LDH release is presented as % of total LDH. IL-1β release is presented as % of unstimulated control and the dotted line represents the unstimulated control **(E)**. Data are presented as mean ± SEM (*n* = 3 independent experiments). Asterisks denote statistical significance compared to respective unstimulated control (*p < 0.05, **p < 0.01, ***p < 0.001).

We found that *NLRP3* (Figure S1) and *AIM2*, but not *NLRP1, NLRC4* or *NLRP6* were expressed at the basal gene level by 5637 cells (data not shown). In order to assess the role of NLRP3 and caspase-1 in UPEC-induced IL-1β release and urothelial cell death, we constructed NLRP3 and caspase-1 deficient cell lines using the CRISPR/Cas9 system. Western blot analysis confirmed decreased caspase-1 (gRNA1 73%, gRNA2 72%) and NLRP3 (> 90%) protein expression compared to epithelial cells transfected with an empty control Cas9 plasmid (Figures 5A,B). CFT073 (MOI 10) induced a significantly lower IL-1β release (Figure 5C) and LDH release (Figure 5D) in caspase-1 -and NLRP3-deficient cells compared to Cas9 control cells after 6 h of stimulation. Caspase-1 deficient cells still had the ability to induce a significantly increased IL-1β and LDH release compared to unstimulated cells, suggesting that additional enzymes could be involved in the maturation of IL-1β and LDH release.

Pharmacological inhibition of caspase with the caspase-1/4 inhibitor Ac-YVAD-CHO significantly reduced IL-1β levels (Figure 5E) and caspase-1 activation (Figure 5F) compared to cells subjected to the vehicle control DMSO. The caspase-3 inhibitor Ac-DEVD-CHO had no effect on IL-1β levels and caspase-1 activation (Figures 5E,F). These results suggest that UPEC-mediated IL-1β release and urothelial cell death *in vitro* is dependent on caspase-1 and NLRP3.

### NLRP3 affects UPEC adhesion and invasion of bladder epithelial cells

**Figure 6:**
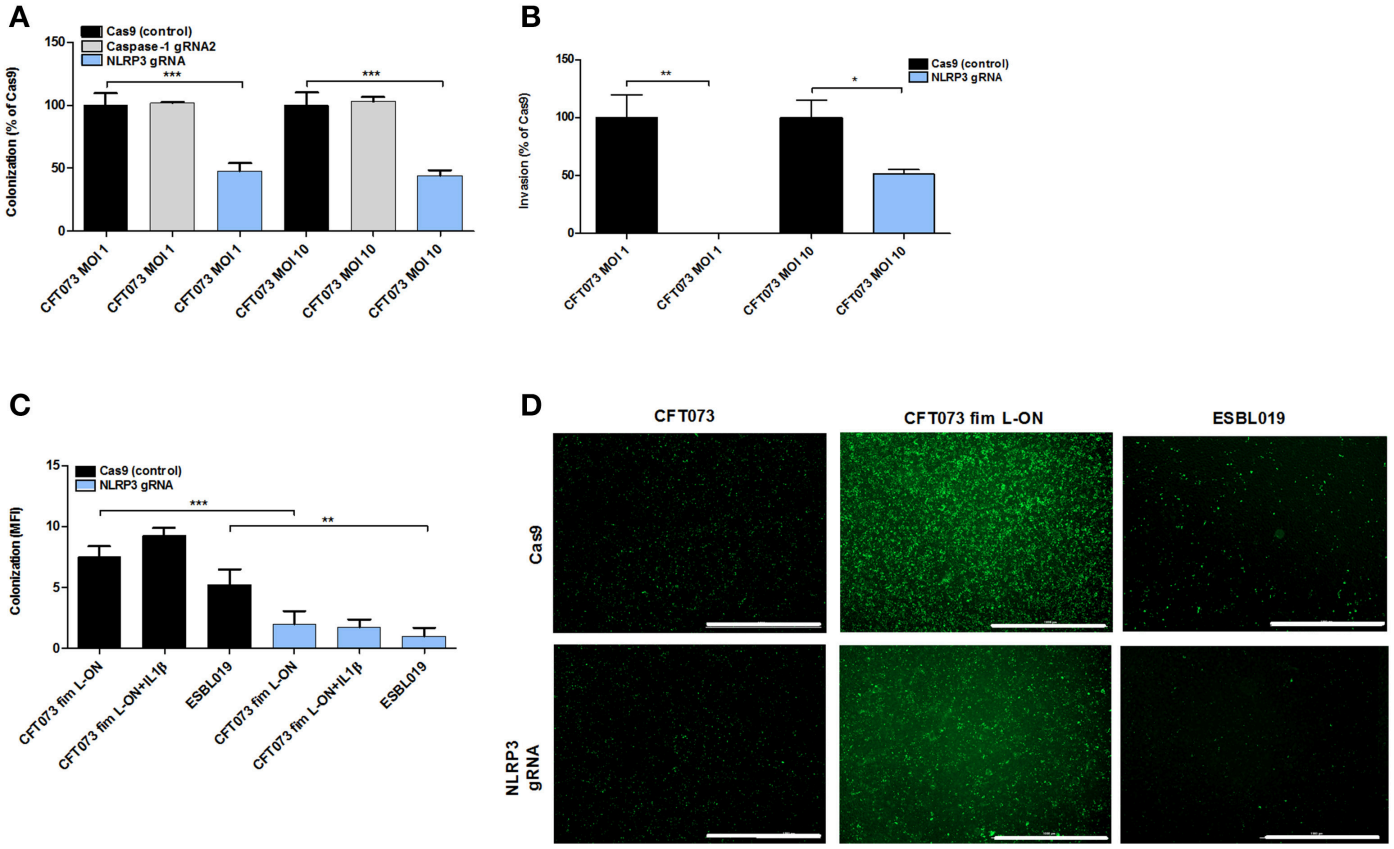
UPEC adhesion and invasion of bladder epithelial cells. Cas9 (control cells), caspase-1 or NLRP3 deficient bladder epithelial 5637 cells were infected with CFT073 (eGFP) **(A,D)**, CFT073 fim L-ON (eGFP) **(C,D)** or ESBL019 (eGFP) **(C,D)** for 2 h **(B)** or 4 h **(A,C,D)** at MOI 1 **(A,B)** and MOI 10 **(A–D)** followed by evaluation of colonization (adhered and intracellular bacteria) and invasion (intracellular bacteria). Cells were pre-incubated with 500 pg/ml IL-1β for 1 h prior to UPEC infection **(C)**. Colonization is presented as percentage of Cas9 colonization **(A)** and as mean fluorescence intensity (MFI) **(C)**. Invasion was presented as percentage of Cas9 invasion **(B)**. Data are presented as mean ± SEM (*n* = 3 independent experiments). Asterisks denote statistical significance compared to respective unstimulated control (*p < 0.05, **p < 0.01, ***p < 0.001). Scale bar: 1,000 μm.

The NLRP3- and caspase-1 deficient epithelial cell lines were stimulated with CFT073 and ESBL019 to assess the involvement of the inflammasome proteins in adhesion and invasion of bladder epithelial cells. A significantly reduced colonization (adhesion and invasion) was found in NLRP3 deficient cells, but not in caspase-1 deficient cells, by CFT073 and ESBL019 at MOI 1 and MOI 10 compared to Cas9 control cells (Figures 6A,C,D). In addition, the ability of CFT073 to invade NLRP3 deficient cells was significantly reduced compare to Cas9 cells (Figure 6B). The reduced colonization of NLRP3 deficient cells was type-1 fimbriae (Figures 6C,D), but not P-fimbriae dependent (data not shown). Pre-incubating the NLRP3 deficient cells with IL-1β did not restore UPEC adhesion (Figure 6B). We continued by evaluating if NLRP3 deficient cells may have a reduced expression of α3β1 integrins and uroplakins, known binding targets for type-1 fimbriae on the surface of bladder epithelial cells (Martinez et al., 2000; Bien et al., 2012). However, the mRNA expression of both integrin β1 and uroplakin 3a were significantly upregulated compared to Cas9 cells after CFT073 stimulation. Integrin α3 and uroplakin 1a were slightly upregulated, but not significantly (Figure S3). Taken together, these results suggest that NLRP3 affects type-1 fimbriae mediated adhesion and invasion of bladder epithelial cells.

### Signaling pathways associated with CFT073-induced caspase-1 activation and IL-1β release

**Figure 7:**
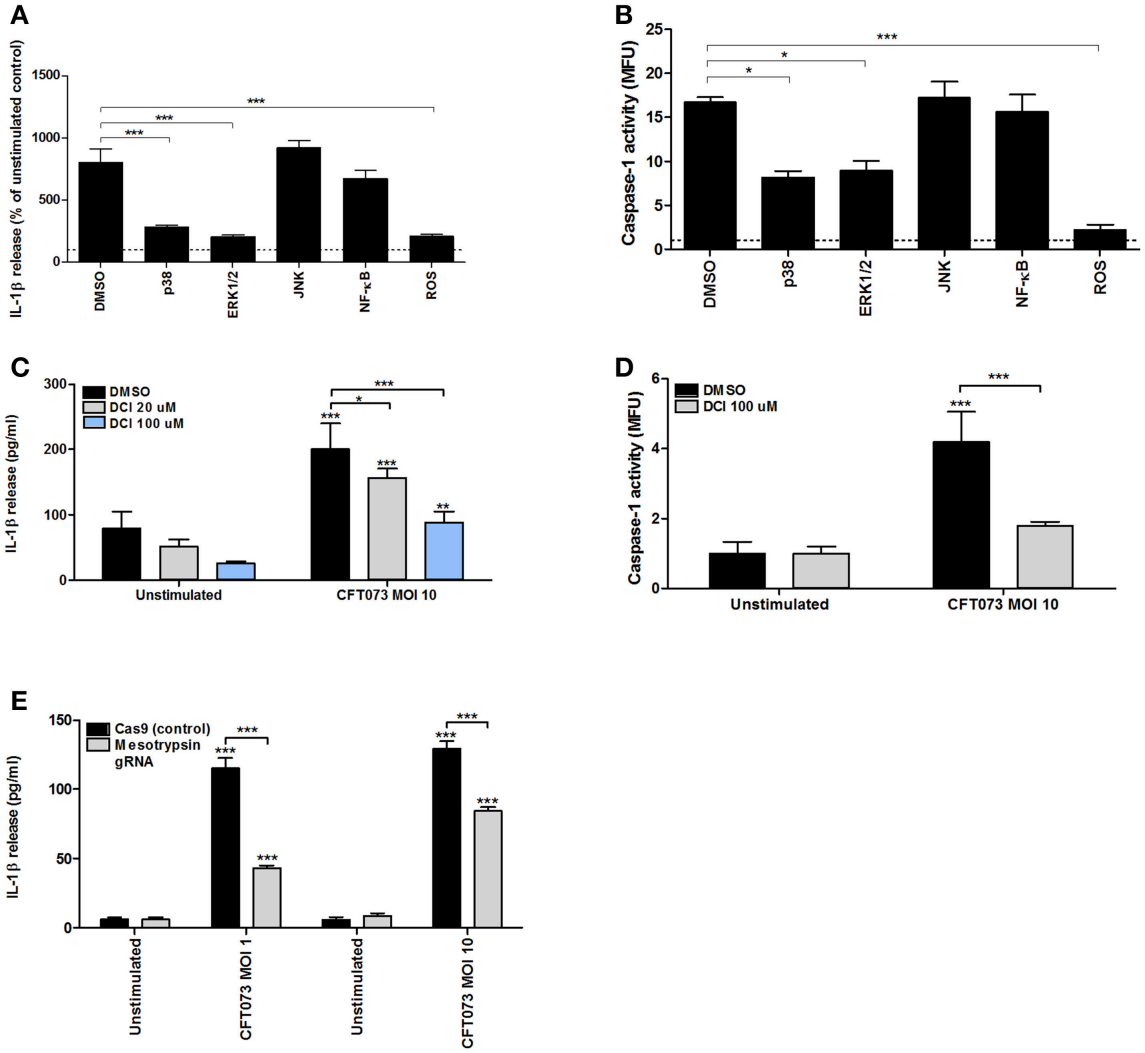
Signaling pathways associated with CFT073-induced IL-1β release and caspase-1 activity. Bladder epithelial 5637 cells were pre-incubated with DMSO (vehicle), JNK inhibitor SP600125 (10 μM), p38 MAPK inhibitor SB203580 (10 μM), ERK1/2 inhibitor PD98059 (10 μM), NF-κB inhibitor BAY 11-7082 (5 μM), reactive oxygen species (ROS) inhibitor diphenyleneiodonium chloride (DPI, 10 μM) **(A,B)**, serine protease inhibitor 3,4-dichloroisocoumarin (DCI) **(C,D)** for 1 h prior to infection with CFT073 at MOI 1 and MOI 10 for 6 h followed by analysis of IL-1β release **(A,C)** and caspase-1 activity **(B,D)**. Caspase-1 activity is presented as fold increase of mean fluorescence units (MFU) compared to unstimulated control cells (dotted line). IL-1β release is presented as % of unstimulated control and the dotted line represents the unstimulated control **(A)**. Cas9 (control cells) or mesotrypsin deficient 5,637 cells were infected with CFT073 for 6 h at MOI 1 and MOI 10 and IL-1β release was measured **(E)**. Data are presented as mean ± SEM (*n* = 3 independent experiments). Asterisks denote statistical significance compared to respective unstimulated control (*p < 0.05, **p < 0.01, ***p < 0.001).

To assess the signaling pathways involved in CFT073-induced caspase-1 activation and IL-1β release in bladder epithelial cells inhibitors targeting p38, ERK1/2, JNK, NF-κB and reactive oxygen species (ROS) was utilized. We found that inhibition of p38, ERK1/2 and ROS, but not NF-κB and JNK, resulted in significantly lower IL-1β release compared to release from cells subjected to DMSO after CFT073 stimulation at MOI 10 for 6 h (Figure 7A). The same pattern was observed for caspase-1 activity (Figure 7B). The cell viability was not reduced by any of the used inhibitors (data not shown). Bladder epithelial cells pre-incubated with the serine protease inhibitor DCI prior to CFT073 released significantly less IL-1β (Figure 7C) and showed reduced caspase-1 activation (Figure 7D) compared to DMSO controls. LDH release from DCI-treated bladder epithelial cells did not differ compared to DMSO treated cells (data not shown).

UPEC have previously been shown to induce activation of the serine protease mesotrypsin though α-hemolysin in bladder epithelial cells (Dhakal and Mulvey, 2012). We therefore tested the hypothesis that mesotrypsin could be the serine protease targeted by the inhibitor DCI. A mesotrypsin deficient 5637 cell line was created and the cells were infected with CFT073 at MOI 1 and 10 for 6 h. Mesotrypsin deficient cells induced a significantly lower IL-1β release compared to the Cas9 control cells upon CFT073 stimulation (Figure 7E). Together these results suggest that UPEC-induced caspase-1 activation and IL-1β release from bladder epithelial cells is mediated by a signaling cascade involving several intracellular kinases, ROS and serine proteases.

## Discussion

The understanding of how UPEC interact with cells of our immune system and how bacteria are manipulating the immune response is becoming more crucial as antibiotic resistance is reducing the treatments options. Recent studies have suggested that the NLRP3 inflammasome and IL-1β are important for the establishment and progression of a UTI (Nagamatsu et al., 2015; Symington et al., 2015; Ambite et al., 2016). This study expands previous knowledge by determining what virulence factors UPEC exploit to modulate inflammasome activation and IL-1β release and also provides insights on the importance of inflammasome activation for UPEC colonization of bladder epithelial cells. In the present study, we showed that the UPEC strain CFT073, but not the non-pathogenic MG1655, induced an increased IL-1β release from bladder epithelial cells accompanied by an increased caspase-1 activation and increased epithelial cell death. We and others have shown that UPEC isolates have the ability to suppress the release of pro-inflammatory cytokines, like IL-6 and IL-8 from bladder epithelial cells, and that the non-pathogenic MG1655 induces higher cytokine release compared to UPEC isolates (Hunstad et al., 2005; Demirel et al., 2013). However, UPEC strains induced higher IL-1β release compared to MG1655, indicating that IL-1β could be beneficial for the bacteria and the progression of the infection. Commensal *E. coli* K-12 strains like MG1655 usually lack the pore forming toxin α-hemolysin (Blattner et al., 1997) that is essential for activation of the NLRP3 inflammasome in bladder epithelial cells (Nagamatsu et al., 2015). Two recent studies performed in mice, present contradictory findings regarding the role of IL-1β. One study found that IL-1β^−/−^ mice do not develop UTI upon infection (Ambite et al., 2016), suggesting that IL-1β produced by the host is crucial for bacterial colonization. However, the other study found that IL-1β could have a protective role and counteract UPEC colonization (Waldhuber et al., 2016). A protective role of IL-1β against a UPEC infection has been suggested earlier as well (Hertting et al., 2003). Infection with other members of the *Enterobacteriaceae* family like *Shigella* (Sansonetti et al., 2000) and *Salmonella* (Raupach et al., 2006) was reported to have lethal effects in IL-1β^−/−^ mice. Hence, the contribution of IL-1β to the host response during UTI is under evaluation.

The processing of IL-1β by activated caspase-1 is well studied (Martinon et al., 2002; Broz and Dixit, 2016; Man and Kanneganti, 2016) and it has been shown that the activation and assembling of the NLRP3 inflammasome and subsequent caspase-1 activation are associated with IL-1β release and epithelial cell death (pyroptosis) in the human urinary tract (Nagamatsu et al., 2015; Ambite et al., 2016). By using caspase-1 -and NLRP3-deficient cell lines or a caspase-1/4 inhibitor, we confirmed the involvement of caspase-1 and NLRP3 in the release of IL-1β from bladder epithelial cells. Western blot analysis revealed that CFT073-stimulation of 5,637 cells resulted in lower protein levels of NLRP3 compared to control. Baroja-Mazo and colleagues have previously shown that the NLRP3 inflammasome can be released extracellular and by that act as a danger signal that amplifies inflammation (Baroja-Mazo et al., 2014). However, western blot analysis of precipitated supernatants did not reveal any extracellular NLRP3 (data not shown). Since NLRP3 has been shown to be degraded by both the proteosomal system (Song et al., 2016) and autophagy (Symington et al., 2015; Jo et al., 2016), degradation may partly explain the lower NLRP3 levels.

Exfoliation of bladder epithelial cells is crucial for the clearance of UPEC, especially as a front-line defense against intracellular UPEC (Nagamatsu et al., 2015). We found that bladder epithelial cells death was associated with activation of NLRP3 inflammasome pathway and caspase-1 activation, in agreement with Nagamatsu and colleagues (Nagamatsu et al., 2015). However, it has previously been reported that UPEC can induce FimH-dependent apoptosis in mice (Mulvey et al., 1998). Thus, the mechanism used by UPEC to induce cell death may vary depending on the isolates virulence profile. It is likely that both apoptosis and pyroptosis are utilized by the immune response to exfoliated bladder epithelial cells during UPEC infection. However, exfoliation can been seen as a double-edged sword by promoting the dissemination of the bacteria into deeper epithelial layers, which may lead to chronic cystitis and recurrent UTI (Mulvey et al., 1998, 2000; Lüthje et al., 2013; Nagamatsu et al., 2015).

UPEC are known to express several virulence factors that can modulate the host immune response to promote persistence in the urinary tract (Bower et al., 2005; Yadav et al., 2010; Bien et al., 2012). However, how these virulence factors interact with the NLRP3 inflammasome pathway in not well characterized. Using P-fimbriae (*pap*), type-1 fimbriae (*fimH*) and α-hemolysin (*hlyA*) CFT073 deletion mutants and a phase-locked type-1 fimbrial ON variant of CFT073 we found that the Δ*hlyA* mutant induced an increased caspase-1 activation and IL-1β release compared to control after 3 h. However, after 6 h, the Δ*hlyA* mutant induced lower IL-1β release and caspase-1 activation compared to control. Importantly, the involvement of α-hemolysin in the activation of caspase-1, release of IL-1β and in epithelial cell death was confirmed in experiments with clinical UPEC isolates and a spontaneously transformed bladder epithelial cell line (HBLAK). A dual concentration-dependent effect of α-hemolysin has been recognized previously in bladder epithelial cells. At a low concentration, α-hemolysin suppresses NF-κB activation and IL-6 secretion from bladder epithelial cells (Dhakal and Mulvey, 2012; Hilbert et al., 2012; Ristow and Welch, 2016), while higher concentrations of α-hemolysin is able to lyse host cells in order for the bacteria to gain access to iron, nutrients and to cross mucosal barriers (Cavalieri et al., 1984; Keane et al., 1987; Ristow and Welch, 2016).

Bacterial adhesion to host cells is crucial for the colonization of the urinary tract. UPEC is known to utilize the type-1 fimbriae for colonizing the bladder and the P-fimbriae for the colonization of the kidneys. The expression of type-1 fimbriae has been shown to enhance UPEC survival, adhesion/invasion of bladder epithelial cells (Rodrigues and Elimelech, 2009) and growth (Eto et al., 2007). In addition, type-1 fimbriae can also modulate mucosal inflammation (Hedlund et al., 2001; Bien et al., 2012). We found that neither the Δ*pap* nor Δ*fimH* mutant altered caspase-1 activation or IL-1β release compared with wild type CFT073 infected cells. These findings indicate that neither of these virulence factors are important for the priming or activation of the NLRP3 inflammasome. The expression of type-1 fimbriae is known to depend on several environmental conditions, such as pH, osmolarity and temperature (Gally et al., 1993; Olsen et al., 1998; Schwan, 2011). Several research groups have failed to detect active type-1 fimbriae expression in UPEC isolated from patient urine (Lim et al., 1998; Roos et al., 2006; Reisner et al., 2014). This can be explained by the recent findings suggesting that planktonic UPEC downregulate the expression of type-1 fimbriae to save energy for growth, and that the type-1 fimbriae is expressed on the sessile population attached to the bladder epithelial cells (Stærk et al., 2016). For this reason, we infected the bladder epithelial cells with the phase-locked type-1 fimbrial ON variant of CFT073 to ensure its expression. Interestingly, we found that CFT073 fim L-ON did not induce caspase-1 activation or IL-1β release from either of the bladder epithelial cell lines even though α-hemolysin expression was intact. It may be speculated that the type-1 fimbriae, needed for early colonization, together with a low concentration of α-hemolysin during the early stages of infection suppresses NLRP3 inflammasome activation and IL-1β release in order for the bacteria to evade host responses and facilitate colonization of the bladder. However, after primary colonization, the levels of α-hemolysin would increase and activate the NLRP3 inflammasome and induce release of IL-1β. This may promote inflammation, which has been shown to drive the progression of the infection (Ambite et al., 2016) and enhance bacterial growth (Porat et al., 1991). Taken together, we have shown that α-hemolysin and type-1 fimbriae have the ability to modulate the activation of the NLRP3 inflammasome pathway, which could contribute to the initial establishment and progression of a UTI.

The activation of the NLRP3 inflammasome is known to be dependent on extracellular priming and induction of intracellular signaling cascades that induces gene expression and post-translational modifications of NLRP3 inflammasome proteins (Broz and Dixit, 2016). We found that p38, ERK 1/2 and ROS, but not NF-κB were involved in regulating the CFT073-induced IL-1β release from bladder epithelial cells. Initially, inflammasome priming was believed to be mediated through a TIR/MyD88/NF-κB-dependent manner (Yang et al., 2012), but it has become evident that the priming cascade is differently regulated depending on the stimuli and the cell type. Intracellular p38 (Moon et al., 2015), ERK 1/2 (Ghonime et al., 2014), ROS (Shio et al., 2015) and NF-κB (Broz and Dixit, 2016) have all been shown to be involved in the priming of the NLRP3 inflammasome in different cell types. Our findings suggest that CFT073 priming of bladder epithelial cells and IL-1β release is p38, ERK 1/2 and ROS, but not NF-κB dependent. These signaling pathways are also involved in IL-6 and IL-8 release from the urothelium cells during a UPEC infection (Tsai et al., 2009; Chen et al., 2011; Demirel et al., 2012). Furthermore, as the caspase-1 deficient cell lines still had the capacity to release IL-1β, we continued to evaluate if additional enzymes could be involved in activating IL-1β. Matrix metalloproteinase-7 (MMP-7) was recently shown to be able to cleave and activate IL-1β in an *in vivo* UTI model (Ambite et al., 2016). In addition, serine proteases have been implicated to be involved in the maturation and release of IL-1β, especially from neutrophils (Karmakar et al., 2012; Schreiber et al., 2012). We found using a serine protease inhibitor, that IL-1β release and caspase-1 activation was reduced compared to the vehicle control. It has previously been shown that α-hemolysin can activate the serine protease mesotrypsin in bladder epithelial cells, which triggers proteolysis of host proteins and subsequent cell exfoliation (Dhakal and Mulvey, 2012). Using a mesotrypsin deficient cell line we demonstrated the involvement of mesotrypsin in the release of IL-1β from bladder epithelial cells infected with CFT073. This suggests that mesotrypsin is involved in the priming step of the NLRP3 inflammasome during a UPEC infection, possibly through α-hemolysin. Taken together, our findings suggest that the activation of the NLRP3 inflammasome pathway by CFT073 in bladder epithelial cells involves several intracellular kinases, serine proteases and reactive oxygen species.

As previously stated, adhesion and invasion of bladder epithelial cells by UPEC is essential for the colonization of the urinary tract (Flores-Mireles et al., 2015). However, the role of NLRP3 and caspase-1 in the adhesion and invasion of bladder epithelial cells has previously not been studied. We found that NLRP3 deficient cells were more resilient against colonization and invasion compared to control cells by a caspase-1 and IL-1β-independent mechanism. Moreover, the reduced colonization in NLRP3 deficient cells was type-1, but not P-fimbriae dependent. The type-1 fimbriae is known to interact with the mannosylated ligands α3β1 integrins (Eto et al., 2007) and uroplakins (Thumbikat et al., 2009) for the adhesion and invasion of bladder epithelial cells. We found that both integrin β1 and uroplakin 3a were upregulated, and not down-regulated, in NLRP3 deficient cells. We speculate that NLRP3 could be involved in the expression of additional, still uncharacterized, mannosylated ligands on the surface of bladder epithelial cells and/or interfere with actin filament rearrangement known to regulate bacterial internalization. Further investigation is therefore needed to elucidate the mechanisms behind the involvement of NLRP3 in the colonization of bladder epithelial cells. These results emphasize that NLRP3 is not only important for the assembly of the inflammasome and maturation of IL-1β, but causes cellular changes that affects host-bacteria interactions.

This study provides insight into the complexity of the host-pathogen interaction during a UTI. The ability of UPEC to modulate the NLRP3 inflammasome by α-hemolysin and type-1 fimbriae, as well as the diverse effects depending on the phase of infection, argues for great adaptability and survival tactics by UPEC. A previously not recognized role for NLRP3 was identified that implicates NLRP3 in regulation of type-1 fimbriae-dependent adhesion and invasion of bladder epithelial cells. Understanding how UPEC modulate our immune system could help us to identify novel therapeutic strategies to obstruct bacterial virulence in order to inhibit pathogenesis and reduce the prevalence of multidrug-resistant bacteria.

## Author contributions

ID, KP, RK, AP, ES, and AB design the study; ID conducted the experiments; ID, KP, RK, AP, ES, and AB analyzed the data; ID, KP, RK, AP, ES, and AB drafted the article.

### Conflict of interest statement

The authors declare that the research was conducted in the absence of any commercial or financial relationships that could be construed as a potential conflict of interest.
