# Supplementary material for: Activation of the NLRP3 Inflammasome Pathway by Uropathogenic Escherichia coli Is Virulence Factor-Dependent and Influences Colonization of Bladder Epithelial Cells
Source: Front Cell Infect Microbiol. 2018 Mar 14;8:81. doi: 10.3389/fcimb.2018.00081 (PMC5890162; doi:10.3389/fcimb.2018.00081)
Supplement: Supplementary file 1 [file Image1.PDF]

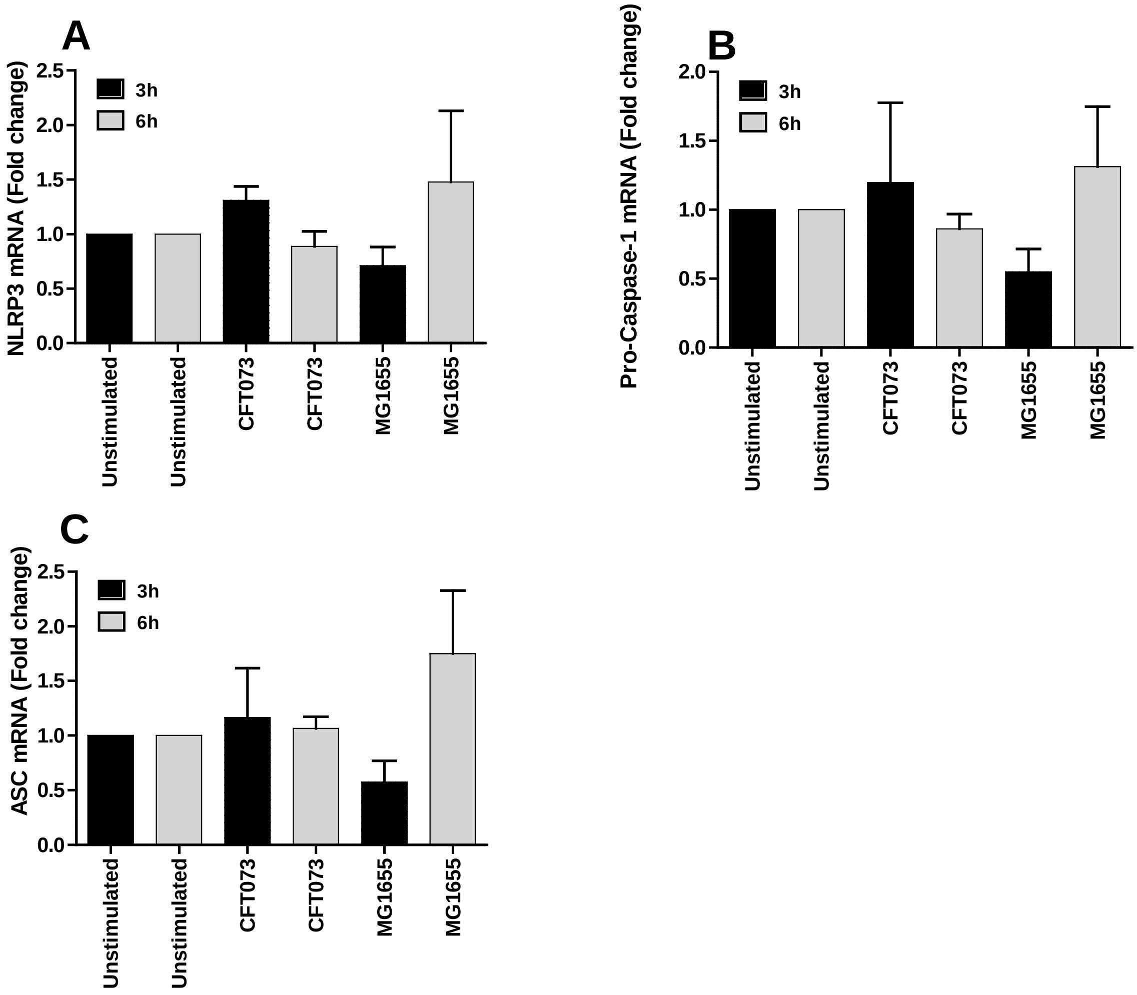

Figure S1: Analysis of mRNA expression. The bladder epithelial cell line 5637 was infected with UPEC strain CFT073 and the non-pathogenic *E. coli* strain MG1655 at MOI 10 for 3 and 6h followed by analysis of NLRP3 (A), pro-caspase-1 (B) and ASC mRNA expression (C). mRNA expression was normalized to GAPDH and is presented as fold change relative to unstimulated controls. Data are presented as mean ± SEM (n = 3 independent experiments).
