# Supplementary material for: Activation of the NLRP3 Inflammasome Pathway by Uropathogenic Escherichia coli Is Virulence Factor-Dependent and Influences Colonization of Bladder Epithelial Cells
Source: Front Cell Infect Microbiol. 2018 Mar 14;8:81. doi: 10.3389/fcimb.2018.00081 (PMC5890162; doi:10.3389/fcimb.2018.00081)
Supplement: Supplementary file 2 [file Image2.PDF]

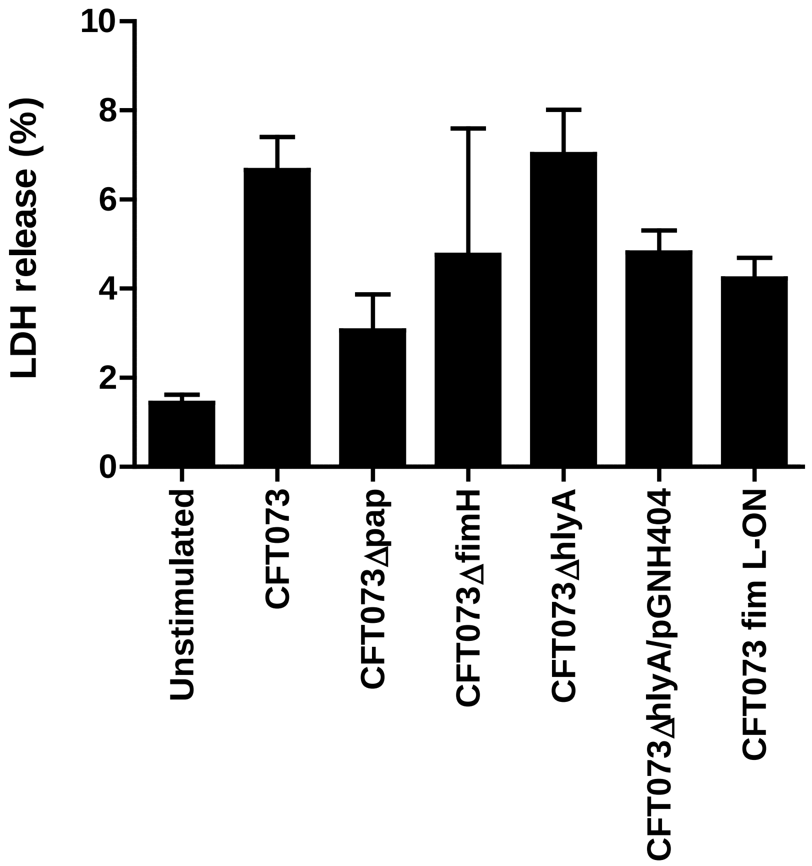

Figure S2: Analysis of LDH release. The bladder epithelial cell line 5637 was infected with CFT073, CFT073Δpap, CFT073ΔfimH, CFT073ΔhlyA, CFT073ΔhlyA/pGNH404 and CFT073 fim L-ON at MOI 10 for 3h and LDH release was evaluated. LDH release is presented as % of total LDH. Data are presented as mean ± SEM (n = 3 independent experiments).
