# Supplementary material for: Activation of the NLRP3 Inflammasome Pathway by Uropathogenic Escherichia coli Is Virulence Factor-Dependent and Influences Colonization of Bladder Epithelial Cells
Source: Front Cell Infect Microbiol. 2018 Mar 14;8:81. doi: 10.3389/fcimb.2018.00081 (PMC5890162; doi:10.3389/fcimb.2018.00081)
Supplement: Supplementary file 3 [file Image3.PDF]

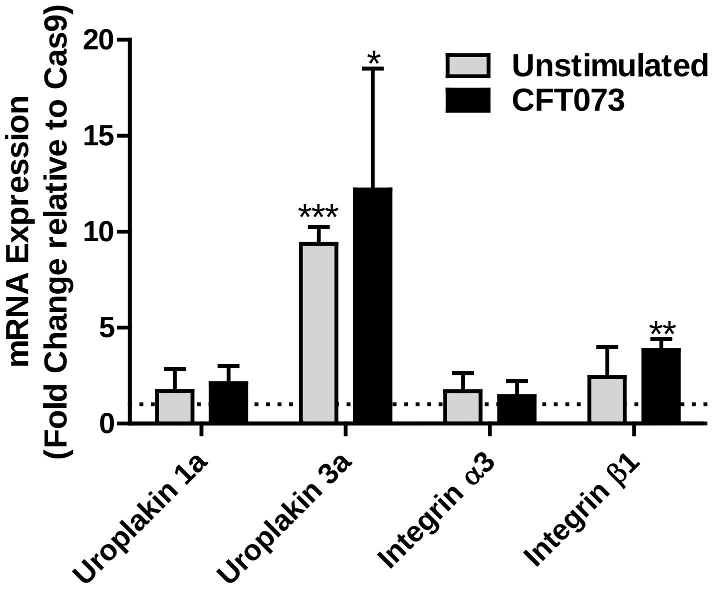

Figure S3: Analysis of mRNA expression. Wild-type Cas9 and NLRP3 deficient 5637 cells were infected with UPEC strain CFT073 at MOI 10 for 6h followed by analysis of uroplakin 1a, uroplakin 3a, integrin α3 and integrin β1 mRNA expression. mRNA expression was normalized to GAPDH and the expression in NLRP3-deficient cells is presented as fold change relative to Cas9 expression (indicated by the dotted line) Data are presented as mean ± SEM (n = 3 independent experiments). Asterisks denote statistical significance compared to respective Cas9 (* $p < 0.05$, ** $p < 0.01$, *** $p < 0.001$).
